# West Riding Hospitals and the Insurance Bill

**Published:** 1911-08-19

**Authors:** 


### WEST RIDING HOSPITALS AND THE INSURANCE BILL.

At the invitation of Mr. Charles Lupton, chairman of the Leeds Infirmary, representatives of thirty-nine hospitals in the West Riding have met to consider the National Insurance Bill now before the House of Commons and its effect on the income of hospitals.

Mr. Lupton presided, and in the course of his remarks said he thought there was no doubt that all sections of their countrymen believed the time ripe for a general effort to be made to provide adequate medical treatment and attendance to all classes, but it was open to serious discussion whether the scheme contained in the National Insurance Bill would produce the desired result. He accepted its general policy, but it was most unfortunate that the Bill should have been framed by those who appeared to be unacquainted with the work of the great voluntary hospitals or unable to appreciate the serious effects it would have on their resources. He reminded them that the great strides made in the science of medicine and surgery had been made almost entirely through the voluntary hospitals, and at considerable expense to the charitable public. The voluntary institutions were the only places where the major portion of the population was able to undergo skilled operations and subsequent nursing and attendance. It was hoped by some of them that the present system of constant operation might in course of time be supplemented by measures of a more preventive nature, and the investigation to arrive at this desirable state of things could only be carried out at institutions where men were working in conjunction with one another and not under isolated conditions. It seemed extraordinary that those connected with hospitals should not have been consulted when the Bill was being framed. The Bill was curiously silent about hospitals, the most direct reference to them being that a patient insured under the Bill should not receive sickness, disablement, or maternity benefit during the time he was an inmate of any workhouse, hospital, asylum or infirmary supported by any public authority or out of any public funds or by a charity. Further, it was enacted that any such benefit otherwise payable to such person should be applied for the relief of his dependents, and if he had no dependents it should be paid to the local Health Committee towards the general purposes thereof. By this clause therefore the hospitals, which undertook the most important and expensive treatment of these patients, were deliberately cut off from receiving any portion of the money raised for the prevention and cure of sickness. Clause 17 permitted an approved society to subscribe to a hospital; it already had the power and did not exercise it to any extent.

With these facts before them, it appeared to those connected with hospitals advisable that steps should be taken to procure the amendment of the Bill, and with that object in view the British Hospitals Association had presented a memorial to the Chancellor of the Exchequer urging that the Bill should be extended to cover treatment in general and special hospitals, and arrangements should be made to recoup all such hospitals for the expenses actually incurred in the treatment of persons insured by the Bill. The deputation was received by Mr. Lloyd George in a very sympathetic manner, but their statement that hospital finances would be injuriously affected was met by the statement that this would not be the case. Mr. Lloyd George quoted the results of the Employers' Liability Act and the experiences of Germany, and after expressing his strong approval of the work done by voluntary hospitals, stated that if he was wrong and they were right the hospitals would not be allowed to suffer.

"It seems to me," added Mr. Lupton, "that it is advisable that we should consider to-day whether Mr. Lloyd George is right in thinking that we shall not suffer by his Bill. His arguments are not convincing to me. It is impossible to compare a great voluntary hospital system like ours with the German system, which is partly on the paying system and partly State-supported."

Alderman B. S. Brigg, of Keighley, moved the following resolution: "That this meeting, representative of thirty-nine hospitals in the West Riding of Yorkshire, having considered the National Insurance Bill now before the House of Commons, and being convinced that a considerable portion of the donations and subscriptions from employees and employers will be discontinued when the payments required under the Bill come into operation, desires to express its complete agreement with the memorial of the British Hospitals Association presented by the deputation on July 25 to the Chancellor of the Exchequer, and wishes to impress upon him the necessity for making provision to recoup the hospitals the cost they incur in treating patients insured under the Bill"; and, having been seconded by Mr. A. E. Barnes, of Sheffield, and supported by several others, it was adopted.

It was agreed to send copies of the resolution to Mr. Lloyd George and the members of Parliament for the West Riding.

